# Catalytically Dead αCaMKII K42M Mutant Acts as a Dominant Negative in the Control of Synaptic Strength

**DOI:** 10.1371/journal.pone.0123718

**Published:** 2015-04-23

**Authors:** Anatoli Y. Kabakov, John E. Lisman

**Affiliations:** Biology Department and Volen Center for Complex Systems, Brandeis University, Waltham, Massachusetts, United States of America; Institute for Interdisciplinary Neuroscience, FRANCE

## Abstract

During long-term potentiation (LTP) of excitatory synapses, Ca^2+^/calmodulin-dependent protein kinase II (CaMKII) is activated by Ca^2+^ influx through NMDA receptors that potentiate AMPA receptor currents by insertion of additional GluR1-containing receptors at the synapse and by increasing AMPA channel conductance, as well as by stimulating structural changes. CaMKII is also involved in the maintenance of LTP and contributes to maintenance of behavioral sensitization by cocaine or amphetamine. Recent studies show that transient expression of catalytically dead αCaMKII K42M mutant after exposure to amphetamine persistently reverses the behavioral effects of the addiction. A suggested interpretation is that this mutant acts as a dominant negative in the control of synaptic strength, but this interpretation has not been physiologically tested. Here we investigate the effect of αCaMKII K42M mutant expressed in single CA1 pyramidal neurons on basal excitatory neurotransmission in cultured rat hippocampal organotypic slices. The mutant caused nearly 50% reduction in the basal CA3–CA1 transmission, while overexpression of the wild-type αCaMKII had no effect. This result is consistent with the dominant negative hypothesis, but there are complexities. We found that the decrease in basal transmission did not occur when activity in the slices was suppressed after transfection by TTX or when NMDA receptors were blocked by APV. Thus, the dominant negative effect requires neural activity for its expression.

## Introduction

Ca^2+^/Calmodulin (CaM)-dependent protein kinase II (CaMKII) has a major function in the long-term potentiation (LTP) of excitatory glutamatergic synapses [1,2]. CaMKII is activated by Ca^2+^ influx through the NMDA-type glutamate receptor and potentiates AMPA-type glutamate receptor (AMPAR) currents by promoting insertion of additional GluR1-containing AMPARs at the synapse [3], by increasing AMPA channel conductance [4,5], and by stimulating structural changes [6]. A specific autophosphorylation site, T286, produces activation of CaMKII that persists after the cessation of Ca^2+^ elevation [7]. Mutation of this site produces a strong inhibition of LTP [8]. Recent work suggests that in addition to having a role in LTP induction, CaMKII, in its association with the NMDA receptors, has a critical role in the maintenance of LTP. Specifically, interfering with this complex after LTP induction can reverse saturated LTP, allowing additional LTP to then be induced [9].

CaMKII has also been demonstrated to have a role in learning and memory. Most dramatically, the mutant (T286A), in which T286 cannot be phosphorylated, produces a profound deficit in memory [8,10]. This deficit is a likely consequence of the observed decrease in LTP induction observed in hippocampal slices containing this mutation. Recent work suggests that CaMKII may also have a role in the maintenance of memory, including drug addiction, which can be considered as a type of memory. Notably, in amphetamine-sensitized rats, transient expression of catalytically dead αCaMKII K42M in the accumbens shell produced a persistent reversal of enhanced amphetamine self-administration [11].

The action of the catalytically dead CaMKII was presumed to work as a dominant negative, and this assumption is critical to the interpretation of the experiments. However, while the assumption that the K42M mutant acts as a dominant negative is reasonable, there has been no experimental demonstration of this. There has been a previous study of the mutant K42R/T286D [6], but the presence of the T286D mutation, which can have an activating function, complicates interpretation of the results. We therefore sought to specifically test whether the K42M mutation alone acts as a dominant negative. Specifically, we used a cultured slice preparation to determine whether K42M CaMKII mutant can produce a decrease in synaptic strength that is not produced by similar expression of wild-type CaMKII.

## Materials and Methods

The Brandeis University Institutional Animal Care and Use Committee (IACUC) approved this study.

### Preparation of organotypic hippocampal slice cultures

Hippocampal slice cultures were prepared from postnatal day 6 (P6)–P7 male Long Evans rats in accordance with Institutional Animal Care and Use Committee (IACUC) guidelines. In sterile conditions, the rats were decapitated, the brain was removed, and 260 μm hippocampal slices were prepared using Vibrating blade microtome Leica VT1000 S (Leica Microsystems Inc.). Sterile, ice-cold modified artificial cerebrospinal fluid (ACSF) saturated with 95% O_2_ and 5% CO_2_ was used during the preparation. Modified ACSF contained the following (in mM): 190 sucrose, 30 D-glucose, 4 KCl, 1 CaCl2, 8 MgCl2, 26 NaHCO3, and 25 HEPES; pH 7.3, osmolality 320 mOsm/kg. Slices were plated on 1.0 μm pore size membrane of six-well plate inserts (Falcon, Cat.# 353102; Corning Inc.) and were incubated in a CO_2_ incubator at 36°C in culture medium containing minimum essential medium (MEM; Sigma, Cat.# M3024) supplemented with the following: 20% horse serum (Sigma), 1 μg/ml insulin, 2 mM GlutaMAX (Invitrogen, Cat.# 35050–061), 23 mM HEPES, 0.2 mM CaCl_2_ (final [Ca^2+^] = 2 mM), 2 mM MgSO_4_ (final [Mg^2+^] = 2.7 mM), 26 mM NaHCO3, 28 mM D-glucose, and 0.5 mM ascorbate. The media was changed three times a week.

### DNA constructs and single-cell electroporation

The green fluorescent protein (GFP)-tagged full-length αCaMKII constructs (wild-type and K42M) used in this study were described previously [12,13]. The GFP contained the A207K mutation to ensure its monomeric state (mGFP). mRFP (mCherry) was used as a bright morphological marker [14]. All constructs were verified by sequence analysis.

Single-cell electroporation (SCE) was performed at 10–14 days *in vitro*, as previously described [15,16]. In brief, slices were placed in a sterile SCE chamber filled with rat Ringer solution containing (in mM) 160 NaCl, 5.4 KCl, 12 MgCl2, 2 CaCl2, and 5 HEPES. Glass micropipettes (4–5 MΩ) were filled with 100 ng/μl mCherry plasmid DNA and 200 ng/μl mGFP-αCaMKII variant (WT or K42M) in “electroporation” Ringer solution containing (in mM): 160 NaCl, 5.4 KCl, 2 CaCl2, 12 MgCl2, 5 HEPES, pH 7.4 (adjusted with NaOH). The tip of the micropipette was placed next to the soma of a CA1 neuron, and a train of 100 of 1 ms pulses of 10V amplitude were delivered at 200 Hz. After transfection by electroporation, hippocampal slices were moved back to the incubator and developed for 1–3 days to allow expression of the proteins. In some experiments, drugs were added to the media immediately after the transfection and were present until the slices were moved to the submersion-type chamber, where the drugs were washed out with ACSF for 1 hour before the electrophysiological experiments. The drugs used include 100 μM DL-2-amino-5-phosphonopentanoic acid (DL-APV; Ascent Scientific, Ellisville, MO) and 1 μM TTX (Sigma). To obtain cells with similar expression level of CaMKII variants in the slices subjected to DL-APV or TTX to the expression in non-treated slices, one, two, or three trains of pulses were delivered to different cells in the same slice during electroporation. In experiments conducted later than 48 h after transfection, the drugs and media were refreshed once. Prior to electrophysiological recording, the drugs were removed by extensive perfusion.

### Imaging

**Fig 1:**
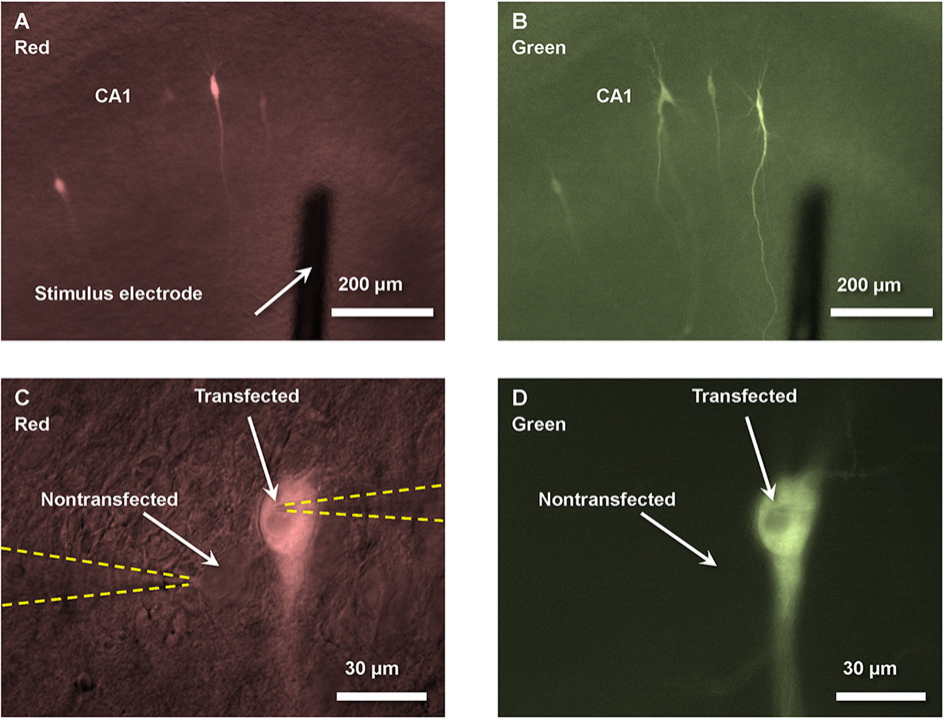
Images of transfected and nontransfected neurons 2 days after single-cell electroporation of organotypic cultured hippocampal slices. **(A)** and **(B)** show multiple neurons in the CA1 region that were transfected with both mCherry (for facilitation of transfected cell localization) and mGFP-αCaMKII K42M variant. Stimulating electrode was positioned 100–200 μm from CA1 cell body layer. **(C)** and **(D)** show neighboring transfected and nontransfected neurons with two patch-pipettes (highlighted by yellow dashed lines) in the whole-cell configuration for simultaneous recordings of EPSCs from these cells. **(A)** and **(B)** show images of mCherry overlapped with DIC image (to show nontransfected cells and electrodes), and (**C)** and **(D)** show images of GFP overlapped with DIC image (see Methods). The images are black and white, but they are colored with red and green to highlight separate filter sets used for different images.

Cells transfected with mCherry cell were visualized with fluorescent differential interference contrast microscopy using an Olympus BX50WI microscope equipped with a 10× water immersion objective (Olympus, UMPlanFl, 10×/0.30 W) with excitation wavelength range 520–550 nm and emission at > 580 nm (Fig 1A and 1C). Fluorescent and differential interference contrast images were overlapped with appropriate illumination intensities to allow simultaneous visualization of transfected and nontransfected cells (Fig 1C). Then, the level of expression of mGFP-αCaMKII constructs was verified using 41025 PSTN GFP Chroma filter set with excitation wavelength at 470 ± 40 nm and emission at 515 ± 30 nm (Fig 1B and 1D). The level of mGFP-αCaMKII expression substantially varied from cell to cell. Therefore, for the experiments we visually selected only those cells, which had comparable distinct GFP fluorescence on a background of differential interference contrast image of the slice at 10× objective using similar levels of illumination. For example, in Fig 1B we chose the far right cell because its strong GFP fluorescence is comparable to fluorescence of other studied cells. The slices, which had a low level of GFP fluorescence in every single transfected cell, were discarded. The electrophysiological experiments were performed under 60× objective (Olympus, UMPlanFl, 60×/0.90 W) (Fig 1C).

### Electrophysiology

Slices at 11–17 DIV were continuously superfused (1.8 ml/min) at room temperature with normal ACSF solution containing (in mM) 124 NaCl, 2.5 KCl, 4 CaCl2, 4 MgCl2, 1.25 NaH2PO4, 26 NaHCO3, 20 dextrose, and 0.05 picrotoxin, 0.003 2-chloroadenosine, balanced with 95% O_2_ and 5% CO_2_, pH 7.4. Glass pipettes of 2–5 MΩ were pulled from borosilicate micropipettes (53432–921; VWR Scientific, West Chester, PA) with a micropipette puller (P-87; Sutter Instruments). The patch pipettes were filled with a solution containing (in mM) 120 Cs-methanesulfonate, 20 CsCl, 10 HEPES, 0.2 EGTA, 4 MgATP, 0.3 Na_3_GTP, and 10 phosphocreatine, pH 7.2, 320 mOsm ⁄ kg. For simultaneous whole-cell recordings, one transfected CA1 neuron and one neighboring nontransfected neuron within 20 μm distance were selected. Both cells were held at -65 mV in voltage-clamp mode, and EPSCs were evoked by stimulating Schaffer-collateral pathway via a platinum-iridium 2-contact cluster electrode (Cat.# CE2C55, FHC, Inc., Bowdoin, ME) placed at 100–200 μm from the cell body layer (Fig 1A). The stimuli were delivered every 6 s with 0.2 ms duration. The intensity of the stimulus (usually between 50 and 300 μA) was adjusted to induce approximately 50 pA α-amino-3-hydroxy-5-methyl-4-isoxazolepropionic acid receptor (AMPAR) excitatory postsynaptic current (EPSC) at -65 mV. In some cases, to reduce probability of polysynaptic responses, we used a smaller amplitude of the stimulus. Series and input resistances were monitored throughout each experiment, and data were rejected if the series resistance varied by 20%. EPSC magnitudes were calculated as average in a 4 ms time window, which started 7 ms after the time of stimulus. Analysis of maximal amplitude of EPSCs in the same time window gave us similar results. The average value of 20 to 50 EPSCs and their s.e.m. are presented. In each experiment, average amplitude of EPSC of a transfected cell was normalized to average amplitude of the simultaneously measured EPSC of a neighboring untransfected cell.

## Results

**Fig 2:**
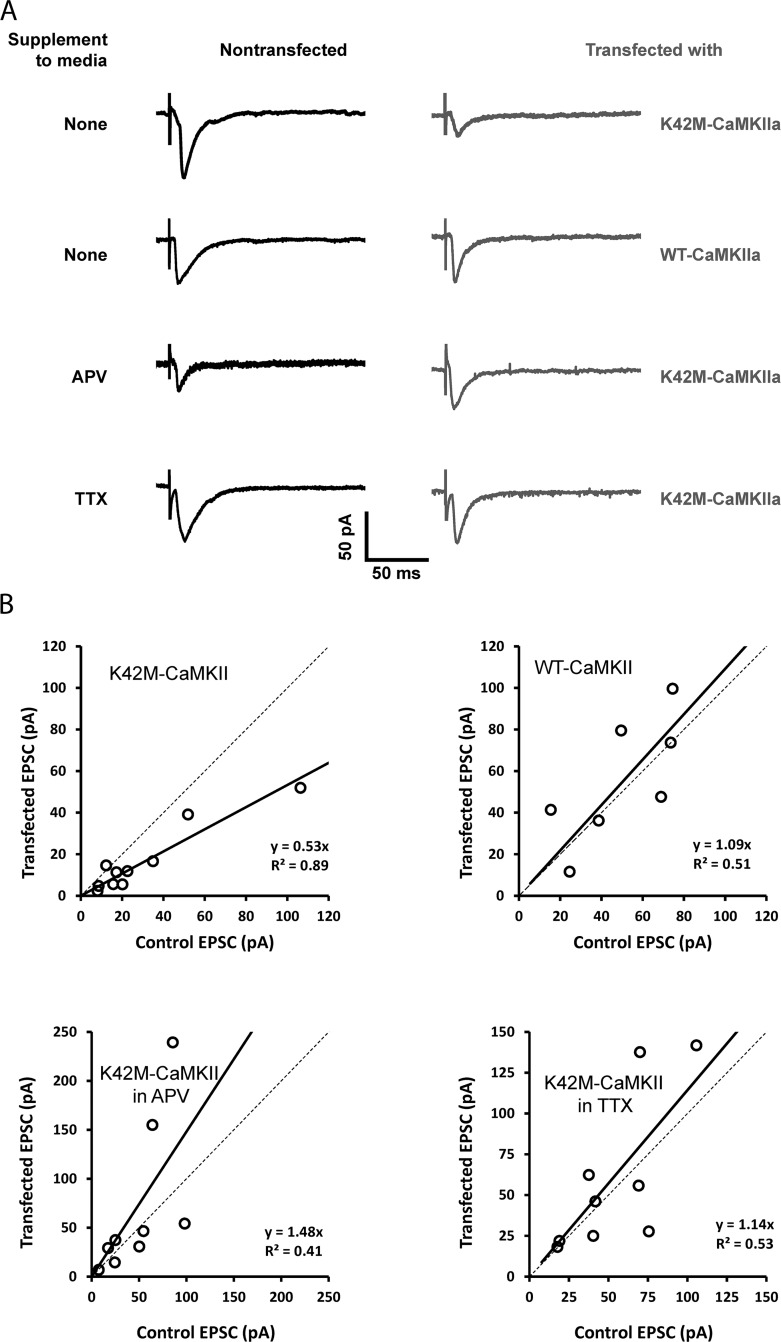
Effect of transfection with ATP binding-impaired αCaMKII K42M mutant under different conditions. **(A)** Example pairs of AMPAR EPSC traces at –65 mV from nontransfected cells and cells transfected with wild type or mGFP-αCaMKII K42M variants in control ACSF or in the presence of 100 μM DL-APV or 1 μM TTX. **(B)** Each dot compares the EPSC in untransfected and transfected cells. Overexpression of wild-type mGFP-αCaMKII has no effect on AMPAR EPSCs in normal media (top right). Overexpression of kinetically dead mGFP-αCaMKII K42M mutant leads to suppression of AMPAR EPSCs in normal media (top left), but effect is blocked by APV or TTX (bottom panels). Thick solid lines correspond to linear fits having the stated slope.

After 10–14 days *in vitro*, CA1 pyramidal cells in organotypic cultured hippocampal slices were transfected (cDNA for a form of αCaMKII and for the morphological marker, mCherry) using single-cell electroporation and were studied 1–3 days later. Transfected cells were visualized by red fluorescence of mCherry (Fig 1A and 1C). The expression of GFP-tagged αCaMKII constructs (K42M or WT) was verified by its green fluorescence (Fig 1B and 1D). We compared the EPSC of transfected cells to nearby untransfected cells as described previously [3,17]. To ensure identical conditions for EPSC recordings from two nearby cells, we employed simultaneous whole-cell recordings from both cells (Figs 1C and 2A). EPSCs were recorded at the holding membrane potential of -65 mV.

**Fig 3:**
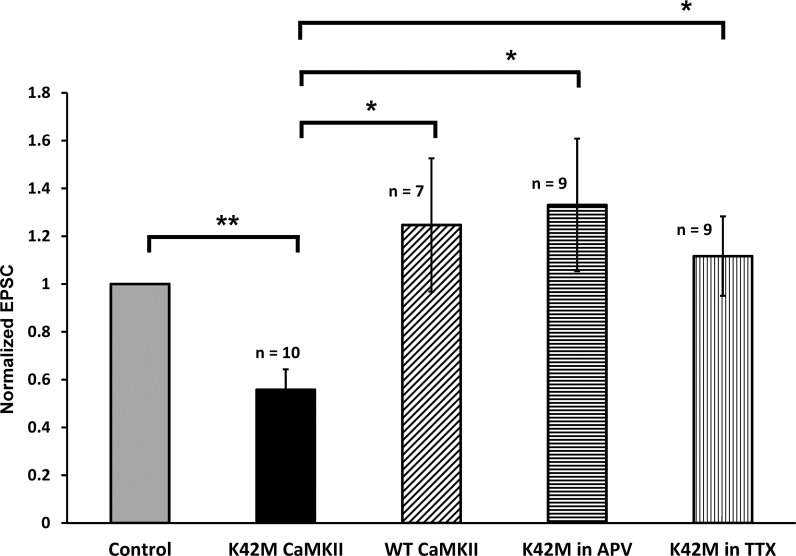
Summary and statistical significance of effects of different forms of CaMKII on EPSC (from data in Fig 2B). Data is presented as the average EPSC normalized to EPSC in untransfected cell. Error bars indicate S.E.M. (*—corresponds to p < 0.05, and **—to p < 0.005).

Initially, we confirmed that transfection with wild-type αCaMKII had no significant effect on the evoked EPSCs relative to EPSCs in nontransfected neurons (Figs 2B and 3), as was previously reported [6]. The apparent increase in the normalized EPSC amplitude in wild-type αCaMKII transfected cells (0.25±0.28, n = 7) is not statistically significant (P = 0.41) but is in the same direction as the increase seen in miniature EPSCs in dissociated hippocampal neuronal cells that over-express wt CaMKII [18,19].

We next tested the effect of transfecting cells with the enzymatically dead mGFP-αCaMKII K42M mutant. In this case, the EPSCs recorded from cells transfected with this mutant had an average amplitude that was 56±9% of the simultaneously recorded EPSCs of nontransfected cells, which was statistically significant (P < 0.005, n = 10) (Fig 3). The duration of the mutant expression in different experiments before the measurements was 1–3 days, but the reduction was already evident at day one and we therefore analyzed all of the data together. The above comparison is mutant compared to nontransfected, but the mutant can also be compared to overexpression of the wild-type αCaMKII. Comparison of normalized EPSCs in the cells transfected with wild-type αCaMKII and in the cells transfected with mGFP-αCaMKII K42M mutant demonstrated significant EPSC reduction in the latter (P < 0.05) (Fig 3). This confirms that the effect is only due to K42M mutation and it is not associated with the transfection itself.

To further characterize the effect of the K42M mutant, we explored the role of spontaneous activity in the slice preparation during the period of transfection. It is known that slices are spontaneously active and that this activity can influence plasticity [20]. We first conducted experiments in which activity was blocked by 1 μM TTX immediately after the single-cell electroporation. After expression was achieved, TTX was removed before measurement of EPSCs, as described in the Methods section. We found that TTX abolished the EPSC reduction produced by the mGFP-αCaMKII K42M mutant (p < 0.05, Fig 3). These results demonstrate the importance of activity in the effect of K42M mutant.

Because effects of spontaneous activity in organotypic cultured hippocampal slices depend on activation of NMDA receptors [20], we next tested whether the effect of mGFP-αCaMKII K42M mutant on the basal excitatory transmission depends on NMDA receptors activation. We found that inhibition of NMDA receptors by 100 μM DL-APV during the mutant expression also abolished the reduction of EPSCs by mGFP-αCaMKII K42M. We observed that the normalized EPSC amplitude in neurons transfected with K42M mutant was equal to 1.33±0.28 (n = 9), which is not significantly different from the neighboring nontransfected control cell EPSC (P = 0.50), but it was significantly different from the mutant EPSC when the slices were incubated in normal ACSF in the absence of DL-APV (P < 0.05, Fig 3). Thus, the inhibiting effect of the expression of mGFP-αCaMKII K42M on basal excitatory transmission depends on NMDA receptors’ activation caused by spontaneous activity in the cultured slices.

## Discussion

Our results show that expression of catalytically dead K42M αCaMKII mutant significantly decreases EPSC amplitudes to 56±9% relative to nontransfected neurons. This is not a result of transfection per se because transfection with wild-type CaMKII had no effect on synaptic strength, consistent with previous results [17]. Our results indicate that the assumption that the K42M mutant acts as a dominant negative is correct. It thus makes sense that this mutant could reverse a form of memory [11].

Our results provide some insight into the mechanism of the dominant negative action of K42M. Specifically, we found that the dominant negative effect requires ongoing synaptic activity because the effect does not occur when the slice is bathed in TTX to block activity or in APV to block NMDA receptors. One possible explanation is in terms of the idea that what is reduced by K42M is a component of baseline transmission. Such a component might be due to maintenance of LTP that had occurred while the animal was still alive [21]. Consistent with this idea, treatment of slices that reduces the CaMKII/NMDAR complex reverses not only LTP [9], but also a component of baseline transmission in pathways where LTP was not induced.

Such a baseline component of the transmission might be affected by K42M in an activity-dependent way because some aspect of maintenance is activity dependent. According to one model of persistence, the ON-state of the CaMKII/NMDAR memory switch is stable against protein turnover because turnover occurs by subunit exchange; a newly inserted unphosphorylated subunit can be phosphorylated by a neighboring active subunit, thereby maintaining the stored information [22]. Recent work shows that activation of CaMKII holoenzyme triggers the exchange of subunits between holoenzymes, including unactivated ones [23]. Thus, one possibility is that K42M mutant affects a process that is only present when activity-dependent protein turnover by subunit exchange occurs.

However, we cannot rule out a very different explanation according to which K42M mutant affects the induction rather than the maintenance process. In this case, the reduction of the EPSC is due to inhibition of spontaneous LTP and thus a change in the balance of LTP and LTD events that occur during the several-day transfection period in slices [20]. However, it seems questionable whether such rapid instances of LTP/LTD occur spontaneously ***in vivo*.**

Finally, it is of interest to note biochemical studies that provide some insight into how the K42M CaMKII mutant might act as a dominant negative. The binding itself of ATP, ADP, or non-hydrolyzable ATP analog (AMP-PNP) to CaMKII enhances CaMKII binding to GluN2B [24]. Furthermore, phosphorylation of CaMKII enhances the binding to GluN2B [25]. Thus, although the mutant can bind to the NMDA receptor and thereby prevent the binding of wild-type CaMKII, it may not form the tight binding necessary for maintenance of synaptic strength.
